# Laboratory automation: a challenge for the 1990s

**DOI:** 10.1155/S146392469400012X

**Published:** 1994

**Authors:** Claude Mordini

**Affiliations:** Rhône-Poulenc, S.A. Courbevoie, Cedex France

## Abstract

There is tremendous pressure on industry and laboratories to develop increasingly complex procucts: for example catalysts, chiral chemicals, drugs and ceramics; conform to regulations; cope with increasingly severe competition; and meet steadily increasing costs. It is difficult, in this situation, to remain productive and competitive. It is vital to be equipped with, and be able to use appropriately, all the suitable methodologies and technologies. Working methods and personnel have to be appropriate. The future depends on three interdependent domains: automation in the broadest sense of the word, instrumentation and information systems. The easy work has already been done. Between 1984 and 1990, it was a question of going from nothing to something; now, it is necessary to increase and optimize.

Therefore, the crucial question is now: ‘how can we go quicker in experimentation and acquire more knowledge, while spending less money?’ One solution is to use all the aspects of automation (robotics, instrumentation, data). Successful laboratory automation depends.on: shortened time to market; improved efficiency/cost ratio; motivation/competence/ expertise; communication; and knowledge acquisition. This paper examines some of the major technological areas of application.


Journal of Automatic Chemistry, Vol. 16, No. 4 (July-August 1994), pp. 125-129

### Laboratory automation: a challenge for the 1990s

**Claude Mordini**
*Rhône-Poulenc, S.A., Courbevoie, Cedex, France*

*There is tremendous pressure on industry and laboratories to develop increasingly complex procucts: for example catalysts, chiral chemicals, drugs and ceramics; conform to regulations; cope with increasingly severe competition; and meet steadily increasing costs. It is difficult, in this situation, to remain productive and competitive. It is vital to be equipped with, and be able to use appropriately, all the suitable methodologies and technologies. Working methods and personnel have to be appropriate. The future depends on three interdependent domains: automation in the broadest sense of the word, instrumentation and information systems. The easy work has already been done. Between 1984 and 1990, it was a question of going from nothing to something; now, it is necessary to increase and optimize.*

*Therefore, the crucial question is now: 'how can we go quicker in experimentation and acquire more knowledge, while spending less money?' One solution is to use all the aspects of automation (robotics, instrumentation, data). Successful laboratory automation depends on: shortened time to market; improved efficiency/cost ratio; motivation/competence/ expertise; communication; and knowledge acquisition. This paper examines some of the major technological areas of application.*

### Applications and results

### *Analysis*

Much has already been published about automation and especially robotization, this paper looks at the kind of added value can be obtained now and in the future. Analysis proceeds along the following lines: sampling/ sample preparation (the most critical steps)/measurement/result/validation/reporting/storage/other use.

### *Objectives*

The aim of the study reported here was to achieve optimum quality of measurements, reliability, safety, flexibility and productivity. The work involved the analysis of pesticide residues in plants and soils.

The system developed involved three robots, one of which was used for chromatographic analysis. There has been a 50% increase in internal capacity for residue analysis and its pay back period was 30 months.

'Industrial—or process—analysis' deserves a special mention. It is an area with important, sometimes insurmountable, difficulties of implementation and in which the stakes are considerable. It is a question of following product development from the raw materials to the finished product, via the manufacturing intermediates, and also of controlling safety and industrial hygiene parameters. Among various industrial analysis methods, a new approach known as 'AT LINE' was developed by the author's company to solve the various problems in 'on line' analysis. The features of AT LINE include: delocalization of analysis to production facilities; analysis is entrusted to manufacturing operators; monoflow or multiflow sequential control with a short response time; very fast feedback on the process; automatic or manual sampling; and automatic feedback of processed data.

The objectives of AT LINE are to guarantee quality, increase productivity, reduce non-conformity costs, to reduce analytical control costs, to act quickly on the process, to optimize cycle times in discontinuous processes.

The benefits include, for hydroquinone/pyrocatechin vanilin manufacturing, a pay back period of two years after investment and a reliability of 0.995.

The future trend is toward: IR and NIR, HPLC and GC, SIA.

NIR between 700 cm-1 and 2500 cm-1 is very popular; and it is used for product identification, and for compliance with specifications through an automatic comparison of the spectrum with those of a library.

In collaboration with Hewlett Packard, Rhône-Poulenc SA developed software for the HPLC technique and the GC technique.

### *Sample preparation*

This is one of the fundamental stages in the process of automatic analysis, process control, product evaluation, screening tests. Reliability and quality of the results are dependent on it. Over 50% of total analysis time is taken by the preparation stage.

The most difficult case is the preparation and handling of solids, for example granules, powders, viscous states. At the author's company, the DAUPHIN system, presented at ISLAR 92, automatically prepares powder samples with automatic weighing for biological screening or for new chemical syntheses. The MISTRAL system, presented at ISLAR 93, prepares the samples in solution for screening.

The general idea is that robots handle objects and no more, and that the stations developed are autonomous, but can be integrated into the information system through an interface.

Another example is sample preparation for the ICP

This paper was presented at the 1993 ISLAR, organized by the Zymark Corporation.

© Zymark Corporation 1994

by mineralization, in particular for polymer analysis. Dissolution is evaluated using a turbidimeter, and if it is not correct, the system activates another cycle.

*Future trends*

Hardware will be improved in the following ways: disposable and more functional laboratory glassware; preventing contamination and loss of product; multiplication of robot arms in order to have more flexible modularity and greater safety; standardization of vials and other accessories; and integrated control systems.

Software and functions will be developed as follows: an automatic taking into account of the corresponding GALP section; management of priority samples; access to information system(s); a single control software for all the modules; access from any workstation, using 'menu driven' procedures, to the properties of a sample, just by interrogating the local server and not the central data base.

*LIMS*

A LIMS is now the typical technology for automated integration in the laboratory environment. It must be based on standards and have an open design to integrate multi-vendor formats, and it requires strong customization. The trend for the future is the integration of CAD tools, of rule-based laboratory control processes.

*Chemical synthesis*

It has been noted that whatever the aim of the synthesis: screening, optimization, new molecule discovery, initial validation of an industrial access pathway, tests usually proceed along the same path and on the same scale (from 0·1 g to 5 g). The general process and timing as follows:

BIBLIOGRAPHY → REACTION → TREATMENT/ ISOLATION/PURIFICATION (50–75% of the time) → ANALYSIS/CHARACTERIZATION

Under these conditions, automation of chemical synthesis is one of the most important goals in pharmaceutical and chemical research, not only from the economic point of view, but also because of the possibility of a very broad screening of reagents and catalysts, in order to direct syntheses towards the most interesting molecules; and finally because it allows the acquisition of knowledge.

There are two complementary and essential approaches: one requires the use of instruments and robots, the other uses the concepts of theoretical chemistry.

For the first method, a three-stage approach was developed at Rhône-Poulenc:

(1) Screening: the aim is to find a new process and the key to success is tightly linked to the number of reactions tried.
(2) Basic understanding: once a new process has been discovered, the reaction must be carefully studied to identify the mechanism, the kinetics, the side products and side reactions, and to understand the influences of 'chemical' parameters.
(3) Optimization: the final stage is performed with the scaling up of the process by the development team. The aim is to reach the industrial process stage as quickly as possible.

A fully integrated application has been developed using two robot arms to perform screening reactions. The chemist can configure up to 30 assays through a user friendly interface. The robot will perform the complete set of assays including the introduction of reagents (solids and liquids), carrying out the reaction, quenching the mixture, treatment, sampling and analysis.

For the basic-understanding training course, the author has developed a fully automated 'micro pilot', which corresponds in fact to the standard chemical reaction vessel (250 ml) which is entirely monitored by an automation and a micro computer.

Future developments will include:

A workstation with integrated reaction and analysis, and also with experimental data management.

Use of knowledge data bases (particularly in catalysis).

More systematic use of drug and chemical design as reaction and reagent screening tools.

All functions will have to be accessible to the user through programmable software and a single interface.

The second approach is the one using computational chemistry and molecular modeling. This field is now widely used in the pharmaceutical industry, but is still little employed in the chemicals industry. The world market, which represented $0.55 billion in 1990, is expected to reach $2 billion by 1996, demonstrating the considerable importance of these methods.

Moecular modeling allows, in the field of the chemical synthesis: the optimization of whole families; the automation of descriptor calculation procedures; the study of reaction pathways and relations with transition states; the integration of spectroscopic and physico-chemical data.

*Information systems*

Information systems are made up of functions and services, data-processing tools, data bases and information. In the scientific field, development has centred on an important application such as the magnagement of chemical structures and of their associated data, at development and corporate scale; these are often carried out in an incoherent and heterogeneous way, depending on the type of organization, user background and because of the lack of standards.

*Current objective*

Each researcher or laboratory manager should have a 'user friendly/menu driven' access to all functions likely to bring added value to their work. This should be carried out automatically from the same workstation with a single interface. In these information systems there are: chemical structures and associated data, chemical reactions, internal reports, electronic mail, LIMS analytical data, molecular modeling and data processing.

Rhône-Poulenc has the following policy: the basic organizational structure is the research centre (RC); all RCs have the same type of information system and the same organization; a light corporate structure ensures general coherence, favours synergies and negotiates contracts for the whole group; and each director supervises the IS in RCs and establishes a two to three year plan with a management committee. The ISs operate will operate on four levels: local, local central, division, corporate; they will use the same basic tools, or compatible tools; they are open to allow the integration of other developments; and they can get access to all data in all four levels within each division, or across division for common domains (structures, screening).

The core of these systems is the chemical structure and the associated data, in particular the biological data obtained from screening and analytical data, very often obtained themselves using robot systems which are thus integrated either functionally or physically through a network.

The major trend for the near future is an increasingly intensive integration of applicatons; these applications must be accessible from an office workstation designed for researchers and will be PC or Macintosch compatible.

*Communication/standards*

Laboratories and especially analysis laboratories are equipped with a heterogeneous equipment, both old and new. The need for standards became unavoidable, as well as the need for communication protocols both for instruments and for automated systems. There are no established standards yet, but work initiated by five american organizations is worth mentioning:

| | |
|---|---|
| ADISS | Analytical Data Interchange and Storage Standards Program |
| AIA | Analytical Instrument Association |
| NIST/CAALS | Consortium of Automated Analytical Laboratory Systems |
| ASTM | American Society for Testing Materials |
| LASF | Laboratory Automation Standards Foundation |

In the field of hardware information system, the 'client-server' architecture is increasingly predominant, both in the analytical process, and in the information systems themselves.

This approach was made possible due to the development of standards for networks and also because the server is a multitask, multi-user machine, thanks to the UNIX system and to protocols such as TCP/IP, MS DOS, X WINDOWS, Microsoft WINDOWS, OSF-MOTIF.

*WINGATES Application*

In the process field, Rhône-Poulenc has developed an in-house communication tool operating with MS-Windows and allowing data exchange with industrial plant, called WINGATES.

*Artificial intelligence*

By artificial intelligence, we refer to a set of methods and techniques that render possible the optimization of a choice, the treatment of questions not arising from a binary choice, pattern recognition; from 'non-linear problems. Several fields can also be distinguished: expert systems, neural networks, fuzzy logic, vision, translation, knowledge management. These are not very widely used yet, but their potential is such that they will certainly become learning players in the automation in the following fields: methods selection, test design, optimization of analytical methods, chemical reaction conditions, elucidation of chemical structures.

*Expert systems*

An expert system is software which reproduces the reasoning of an expert faced with a given situation. It cannot deal with a situation that is not described in the system.

Most expert systems are based on more or less complex rules, with variables or identified facts, with or without actions. Prospects for expert systems at Rhône-Poulenc include: increasingly specific and small systems (less than 100 rules); development of systems which are increasingly directed towards use; connection of several expert systems, and integration.

*Experimental design*

Experimental design is at the core of experimental research methodology. The aim is to propose the best strategy in order to seek the critical factors, to reduce experimental cost, to improve on a property, to remove a defect, to find a model. Experimental design is a tool intended to help ask the right questions, to reduce the number of experiments, if possible, to carry out more significant experiments, to get the most complete information set whatever the strategy, to analyse results. The return on investment is usually significant.

*Neural network*

A neural network is built on the interconnections of elementary computing units; it is organized in layers, each element of which is connected to all those of the following layer. Each link, or synapse, weighs the information which it receives from the emitting neuron in the previous layer. A set of data is thus entered into the network and the solutions obtained are compared; differences are used to adjust the weighing of network connections. A neural network is not intended for calculations and must not be used to solve a problem for which there already exists a good algorithm. However, they are very useful for non-linear phenomena. Their efficiency lies in the fact that signals are treated in parallel; there is no hierarchy between the network units. It is estimated that their application is distributed among: biochemistry 35% (QSAR, fermentation [prediction of the variables of the in-line process], sequences), organic synthesis 5%, chemical engineering 20%, and 35% in the field of analysis.

*Knowledge management*

There are three forms of knowledge: know-how, conceptual knowledge, knowledge of the field. Knowledge management aims at expressing/recovering/storing/exploiting/enriching/distributing all three types of knowledge.

In concrete terms, three types of problems arise:

I want an answer to a problem
→ Expert system

I want to know what has been done about a subject
→ Documentary data base

I want relevant basic knowledge
→ Knowledge-based
→ management system

The conservation of know-how, the search for information, the avoidance of redoing, training, increase in productivity, shared knowledge (assistance for licensing processes) are the important issues.

### Discussion

*Impact on personnel and organization*

Much has been said over the past few years on this subject, but mainly on robotics aspects. It is clear that more severe competition and the new demands from companies in this uncertain environment require: adaptability/speed/flexibility/anticipation. But it is essential that further thought, extending to all the fields of R & D and of the laboratory in particular, must be given to these problems.

The tremendous developments in computing technologies allow this adaptation with genuine re-engineering. The organization and the human environment will have to be changed as a consequence of re-engineering.

*Personnel*

We know that management requires: rapid delivery, high quality, acceptable cost. Three approaches were tested at Rhône-Poulenc: first, to have specialists with a double background in the unit. Second, to associate much more these specialists with the researchers; this requires the creation of multi-disciplinary teams with a common objective. Third, to regard automation specialists as researchers and to allow them progress along the same scale.

*Organization*

The various fields of automation must be formed into a service or department employing the experts from each field. In research departments, the presence of correspondents with a double background must be encouraged. Finally, organization into projects makes it possible to meet as well as possible the three criteria: rapid delivery, high quality, acceptable cost.

*Laboratory design*

Automation technologies are an element of laboratory re-engineering and and require a different laboratory design. The operator workplace is changing, because instrumentation is taking more and more space with its peripherals.

The laboratory becomes flexible and the work surface increases and becomes organized so as to minimize operator movements and reduce risks of error.

Robot, automatism, instrument are the backbone on which modules are grafted; the whole being supervised by a workstation.

*Benefits of laboratory automation*

As we have just seen, laboratory automation is an increasingly complex and integrated set of major functions and information systems.

Consequently, it becomes more difficult, and sometimes nearly impossible, to measure the benefits derived, due to the lack of sufficiently reliable detector.

It is not sufficient to assert that automation improves productivity, reduces the duration of RD, reduces costs, reduces the time to market, allows the development of new products.

When it comes to robotics, it is easy to assess the benefit expressed in ROI concerning quantitative aspects and in terms of added value for the qualitative aspect. Thus, for synthesis screening, the pay back period is 18 months and the number of patents taken is multiplied by three.

In chemical synthesis, the system already described allowed the optimization of the catalyst for the product used in the reaction. Moreover, the system makes it possible to have more claims in patents, the number of registered patents being usually twice as important.

This ease of evolution is due to the fact that once a robot application is finished, it is stabilized for a certain time in its current operation.

This is not the case for the other functions examined above: they are constantly evolving and developing. Therefore, they require annual investments and additional operating expenses, and this is particularly true for data-processing technologies. In this respect, data analysis and experimental design deserved special mention, as in many cases they allowed a two or three-fold reduction in the number of experiments. These two fields have benefited enormously from the dramatic development of data-processing technologies, computers and software; however, although their qualitative impact is acknowledged, their quantitative impact is very difficult to assess. On the other hand, molecular modeling comes very early in the process of pharmaceutical research. However, it acts as a screening tool and now also as an explanatory and knowledge acquisition tool.

Unfortunately, this field is not very popular and some research managers are still not convinced, especially in the chemical industry.

### Conclusion

Laboratory automation in research and manufacturing is a very fruitful approach to control competition and an unstable economic environment.

It is a very significant source of costs, although it is seldom possible to calculate a real ROI; rather, the added value has to be identified and the qualitative benefit assessed.

Hardware and software must be both developed simultaneously using a global approach. To achieve this, true laboratory re-engineering is necessary through progressive integration of the applications; the availability of standards; simpler, but more optimized instruments with regard to their tasks, in particular faster and smaller robots; wider use of new technologies.

However, this requires (in order of priority): specialists with several areas of expertise; educating the management, particularly directors and VPs; adequate organization of RD teams in this field, but also of the manufacturing laboratories' teams.

Success depends on men, markets, organization, and not just technologies.

### Acknowledgements

The author wishes to thank the following people for their help: Dr J. R. Desmurs, Mr L. Drugeault, Mr P. Monnet and Mr J. Vial.

